# Ex Vivo Model to Evaluate the Antibacterial and Anti-Inflammatory Effects of Gelatin–Tricalcium Phosphate Composite Incorporated with Emodin and Lumbrokinase for Bone Regeneration

**DOI:** 10.3390/bioengineering10080906

**Published:** 2023-07-31

**Authors:** Wen-Ling Wang, Yuan-Man Hsu, Meng-Liang Lin, Shih-Shun Chen, Yi-Hui Lai, Chiung-Hua Huang, Chun-Hsu Yao

**Affiliations:** 1 School of Post-Baccalaureate Chinese Medicine, China Medical University, Taichung 40202, Taiwan; supercocono1@mail.cmu.edu.tw; 2 Department of Chinese Internal Medicine, China Medical University Hospital, Taichung 40202, Taiwan; 3 Department of Chinese Medicine, China Medical University Hospital Taipei Branch, Taipei 11449, Taiwan; 4 Department of Animal Science and Technology, Tunghai University, Taichung 407224, Taiwan; yuanmh@thu.edu.tw; 5 Department of Medical Laboratory Science and Biotechnology, China Medical University, Taichung 40202, Taiwan; mllin@mail.cmu.edu.tw; 6 Department of Medical Laboratory Science and Biotechnology, College of Medical and Health Science, Asia University, Taichung 41354, Taiwan; dr.chen3693@gmail.com; 7 Department of Biomedical Imaging and Radiological Science, China Medical University, Taichung 40202, Taiwan; n6772000@yahoo.com.tw; 8 Department of Medical Laboratory Science and Biotechnology, Central Taiwan University of Science and Technology, Taichung 40601, Taiwan; 9 School of Chinese Medicine, China Medical University, Taichung 40202, Taiwan; 10 Department of Biomedical Informatics, Asia University, Taichung 41354, Taiwan; 11 Biomaterials Translational Research Center, China Medical University Hospital, Taichung 40447, Taiwan

**Keywords:** tricalcium phosphate, emodin, lumbrokinase, antibacterial, anti-inflammatory

## Abstract

Tricalcium phosphate (TCP) has gained attention due to its interconnected porous structures which promote fibrovascular invasion and bony replacement. Moreover, when gelatin is added and crosslinked with genipin (GGT), TCP exhibits robust biocompatibility and stability, making it an excellent bone substitute. In this study, we incorporated emodin and lumbrokinase (LK) into GGT to develop an antibacterial biomaterial. Emodin, derived from various plants, possesses antibacterial and anti-inflammatory properties. LK comprises proteolytic enzymes extracted from the earthworm Lumbricus rubellus and exhibits fibrinolytic activity, enabling it to dissolve biofilms. Additionally, LK stimulates osteoblast activity while inhibiting osteoclast differentiation. GGT was combined with emodin and lumbrokinase to produce the GGTELK composite. The biomedical effects of GGTELK were assessed through in vitro assays and an ex vivo bone defect model. The GGTELK composite demonstrated antibacterial properties, inhibiting the growth of *S. aureus* and reducing biofilm formation. Moreover, it exhibited anti-inflammatory effects by reducing the secretion of IL-6 in both in vivo cell experiments and the ex vivo model. Therefore, the GGTELK composite, with its stability, efficient degradation, biocompatibility, and anti-inflammatory function, is expected to serve as an ideal bone substitute.

## 1. Introduction

Bone graft procedures are increasingly utilized in various medical fields, including traumatology, infection treatment, oncology surgery, spine surgery, and revision arthroplasty. The sources of bone could be from various origins, such as autografts (patient’s own bone), allografts (donor bone from another individual), or synthetic substitutes [1]. Autologous bone grafts can cause harvest-related complications and are limited in patient volume; allografts have emerged as an alternative [2]. However, this has resulted in a shortage of donor tissue typically used in these bone reconstruction surgeries. Consequently, the demand for bone substitutes with different compositions, mechanical strengths, and functional biological mechanisms has been on the rise. Besides possessing biomechanical stability and degrading within an appropriate timeframe, an ideal bone substitute should also exhibit osteoconductive, osteogenic, and osteoinductive properties while being biocompatible and eliciting no adverse inflammatory responses [2,3,4].

Orthopedic implants are susceptible to infections, primarily caused by microorganisms that form biofilms and adhere to the implant surface [5,6]. Bacterial pathogens in the biofilm state show remarkable resistance to external attacks such as antibiotics, chemicals, and disinfectants [7]. Moreover, they display insensitivity to drugs and host immune responses when residing within cells [8]. The adverse effects of antibiotic therapies include systemic toxicity, allergy, gastrointestinal problems, and the emergence of bacterial resistance. Therefore, the development of antimicrobial composites to prevent bacterial adhesion and colonization is crucial in the field of biomaterials [9,10].

Various approaches have been employed to develop antimicrobial composites, including the incorporation of antimicrobial agents [11], coating techniques [12,13], and immobilization in biomaterials [14,15]. This study focused on investigating a biodegradable composite made from genipin-crosslinked gelatin mixed with tricalcium phosphate (TCP) ceramic particles (GGT). TCP has garnered attention as a synthetic bone graft substitute due to its interconnected porous structures which facilitate fibrovascular invasion and bony replacement [16,17]. The addition of gelatin to TCP resulted in GGT, which exhibited strong biocompatibility and osteoconduction, making it a promising bone substitute [18,19]. Furthermore, lumbrokinase (LK) and emodin were incorporated into GGT to create an antibacterial biomaterial.

Treating biofilm-related infections using antibiotics poses significant challenges, thus necessitating the use of biofilm disruptors as the primary approach. LK, a fibrinolytic enzyme, was isolated from the earthworm *Lumbricus rubellus* [20]. It can dissolve the fibrin or converts plasminogen to plasmin by inducing endogenous tissue plasminogen activator (t-PA) activity [21]. The formation of *S. aureus* biofilms has been reported to rely on the coagulase-catalyzed conversion of fibrinogen to fibrin [22]. LK can dissolve biofilms through its fibrinolytic activity and has been extensively applied as an implant coating to combat biofilms [23,24].

Emodin is a naturally occurring anthraquinone derivative isolated from the roots, barks, and other parts of numerous plants, molds, and lichens. It serves as the active ingredient in several Chinese herbal medicines, including rhubarb and Polygonum multiflorum. Emodin exhibits a range of beneficial effects, including diuretic, vasodilatory, antibacterial, antiviral, antiulcer, anti-inflammatory, and anticancer properties [25]. In a study conducted by Dr. Li, emodin demonstrated antibacterial activity by disrupting cell membrane integrity, increasing membrane permeability, and influencing membrane protein conformation [26]. Additionally, emodin is believed to possess anti-inflammatory properties by modulating the production of IL-6 and TNF-α [27,28,29].

The objective of this study is to develop an ideal bone substitute biomaterial with antibacterial, anti-inflammatory, biodegradable, and biocompatible properties. Biodegradable composites comprising genipin-crosslinked gelatin mixed with tricalcium phosphate were loaded with lumbrokinase and emodin (GGTELK). *Staphylococcus aureus* (*S. aureus*) was chosen to evaluate the antibacterial effect of the GGTELK composite. Additionally, biocompatibility and osteocyte activities were assessed using colorimetric assays, total alkaline phosphate (ALP), and total tartrate-resistant acid phosphatase (TRAP) assays. The anti-inflammatory effects of the GGT composite were measured using an IL-6 ELISA kit. In vitro cell experiments were followed by the utilization of an ex vivo bone defect model to evaluate the antibacterial and anti-inflammatory effects of the GGTELK composites.

## 2. Material and Methods

### 2.1. Cell Culture

The human osteoblast-like cell line MG-63 (BCRC no. 60279, Food Industry Research and Development Institute, Hsinchu, Taiwan) was cultured in Dulbecco’s Modified Eagle’s medium (DMEM; Gibco, Grand Island, NY, USA), 10% fetal bovine serum (FBS; Gibco), and 1% penicillin/streptomycin (Gibco). In addition, the osteoclast murine monocyte/macrophage RAW 264.7 cells (BCRC no. 60001, Food Industry Research and Development Institute) were cultured in a growth medium of α-MEM with 12% FBS and 1% penicillin/streptomycin (Gibco). Both cell lines were grown at 37 °C under 5% CO_2_ in air. MC3T3-E1 is a clone from a preosteoblast cell line that derived from newborn mouse calvaria. The cell line grew in Modified Eagle’s medium (MEM, Gibco-BRL, Rockville, MD, USA) containing 10% calf serum (FBS, Gibco, Grand Island, NY, USA) and 1% penicillin/streptomycin (Gibco, MD, USA) cultivated in a 37 °C, 5% CO_2_ incubator.

### 2.2. Antibacterial Assay

#### 2.2.1. Turbidimetric Assay

A turbidimetric assay was developed for the quantitation of viable bacterial densities. *S. aureus* at log 5.6 CFU/mL density were treated with 8 µg/mL emodin and 1 µg/mL LK, respectively. After 24 h at 37 °C, the cell densities of *Staphylococcus aureus* (ATCC25923) were determined by turbidimetric measurement (optical density at 600 nm) with reference to the standard curve.

#### 2.2.2. Microtiter Plate Biofilm Formation Assay

A single colony of *Staphylococcus aureus* was removed from the tryptic soy (TS) plate medium into 3 mL of tryptic soy broth medium using an inoculum stick. After overnight incubation at 37 °C, the cell suspension was diluted to 1 × 10^5^ colony-forming units (CFU)/mL with TS broth. Then, 1 mL of the diluted cell suspension was incubated in a 24-well flat-bottomed polystyrene microtiter plate at 37 °C for 24 h. After removing non-adherent cells, the plates were washed twice with deionized water and allowed to air dry. Subsequently, 1 mL of 0.1% crystal violet solution was then added to all wells. After 20 min of incubation, the excess crystal violet was discarded and the plates were washed twice and air-dried. Finally, cell-bound crystal violet was dissolved in 33% acetic acid and 200 µL of dissolved solution was transferred to a 96-well plate, and biofilm growth was measured at an optical density of 595 nm (O.D. 570 nm) using a microplate reader (Multiskan, Thermo Labsystems, MA, USA).

### 2.3. Cell Viability Assay—MTT Assay

Cells were plated at a density of 10,000 cells per well and incubated for a period of two days. Following the two-day incubation, the culture medium was replaced with 10 µL of MTT solution (5 mg/mL) and 100 µL of fresh culture medium per well. The plate was then incubated at 37 °C for 4 h, allowing for the formation of insoluble formazan crystals. Afterward, the solution was aspirated and 100 µL of acidic isopropyl alcohol (0.04 M HCl in isopropyl alcohol) was added to each well in order to dissolve the crystals. The plate was gently shaken for several minutes, and the concentration of dissolved crystals and 200 µL of the dissolved solution were transferred to a 96-well plate and measured using an enzyme-linked immunosorbent assay (ELISA) reader (uQuant; Bio-Tek Instruments, Inc., Sunnyvale, CA, USA) at a test wavelength of 570 nm, with a reference wavelength of 650 nm. The results were expressed as a percentage of relative cell viability compared to the control, and the experiments were performed in triplicate.

### 2.4. Analysis of ALP (Alkaline phosphatase) Activity

After culturing for two days, the medium was substituted with 20 µL of 0.1% Triton X-100 (Sigma, St. Louis, MO, USA) per well and incubated at room temperature for 5 min to induce cell lysis. Within 1 min, 100 µL of a commercially available ALP assay kit (Procedure No. DG1245-K; Sigma) was added to each well. The absorbance at 405 nm, resulting from the generation of p-nitrophenol, was continuously measured for a duration of 30 min using an ELISA reader. The rate of change in absorbance directly corresponded to the overall activity of alkaline phosphatase (ALP). The outcomes were expressed as a percentage of relative total ALP activity compared to the control group, obtained by dividing the total ALP activity of each lumbrokinase solution by that of the control group. The experiments were conducted three times under each specified condition [30].

### 2.5. Analysis of TRAP (Tartrate-Resistant Acid Phosphatase) Assay

After six days of culturing, TRAP activity was evaluated by measuring the amount of acid phosphatase (ACP) released from cells into the medium using a commercial ACP assay kit (Procedure No. 435, Sigma). In brief, 30 µL of the culture medium was mixed with 100 µL of ACP reagent. Absorbance at 405 nm by the produced p-nitrophenol was measured for 30 min using an ELISA reader. The change in absorbance rate was directly proportional to the total TRAP activity. Therefore, results were expressed as relative total TRAP activity (% of control), obtained by dividing the total TRAP activity of each lumbrokinase solution by that of the control group. Under each condition, the experiment was performed three times.

### 2.6. IL 6 ELISA Assay

Assays for IL 6 were carried out using a commercially available enzyme-linked immunosorbent assay (ELISA) kit. All assays were conducted following the manufacturer’s instructions. The concentration of IL 6 was calculated with reference to a standard curve constructed using the recombinant cytokine provided with each kit.

### 2.7. Preparation and Characterization of Porous GGTELK Composites

#### 2.7.1. Preparation of Porous GGT and GGTELK Composites

GGT composites were synthesized following a previously described method [16]. Initially, a homogeneous solution of gelatin (porcine gelatin with a Bloom number of 300, Sigma) was prepared by dissolving it in deionized water at 75 °C using a water bath. As the gelatin solution was cooled to 40 °C, a genipin solution (0.5 wt%, Challenge Bioproducts, Yunlin, Taiwan) was added to induce crosslinking of the gelatin at a constant temperature. The gelatin–genipin mixture was stirred for 2 min, and then β-tricalcium phosphate (β-TCP) particles with grain sizes ranging from 200 to 300 μm (Merck, Darmstadt, Germany) and sieved sodium chloride particles with sizes between 420 and 590 µm (used as a porogen) were added to the mixture to ensure homogeneity. Before use, the salt particles were dried in an oven at 170 °C for 4 h. The weight ratios of gelatin to β-TCP and salt to the gelatin/β-TCP/genipin composite were 1:3 and 3:1, respectively. Vigorous stirring led to an increase in viscosity of the mixtures, which were then poured into plastic dishes and solidified at room temperature for 24 h. The solidified composites were subsequently cut and shaped into cylindrical specimens of specific dimensions. To remove the salt particles, the composites were immersed in deionized water for 24 h, with water being replaced three times during this period. Finally, the samples were frozen at −80 °C for 24 h and freeze-dried for an additional 24 h to obtain porous GGT composites. The dried cylindrical composites had a diameter of 7 mm and a thickness of 5 mm. A similar procedure was followed to prepare GGTELK composites, where lumbrokinase and emodin solutions were added to the gelatin–genipin mixture at 40 °C, followed by the addition of β-TCP and sodium chloride to form porous GGTELK composites.

#### 2.7.2. Drug Release Assay

One-milligram specimens of GGTE were individually immersed in 1 mL of distilled water for varying durations of 0.5, 1, 1.5, 3, 6, 12, 24, and 48 h. Subsequently, each specimen was subjected to UV-VIS spectroscopy at a wavelength of 530 nm for analysis of emodin.

#### 2.7.3. Degradation Assay

To each weighed GGTELK sample, one milliliter of PBS was added; the samples were subsequently incubated for durations of 1, 3, 6, 14, and 28 days. After incubation, each sample was carefully blotted with filter paper, followed by lyophilization and reweighing. The weight loss percentage (%) was determined using the formula (Wi–Wd)/Wi × 100%, where Wi represents the initial weight of the specimen in its dry state and Wd corresponds to the weight of the specimen in its dry state after immersion in PBS for a specific time period.

### 2.8. Evaluation of GGTELK Scaffold Using an Ex Vivo Bone Defect Model

Calvarias were obtained from neonatal Wistar rats at approximately 4 days old. The animal use protocol (protocol ID: CMUIACUC-2019-057) was approved by CMU’s Institutional Animal Care and Use Committee (IACUC). The rats were euthanized with an overdose of Pentothal (0.5–1.0 mL), and the calvaria was carefully dissected. Both the endocranial and extracranial periosteal tissues were completely removed to minimize fibroblastic impurities. The excised calvaria was immediately immersed in PBS. Using a sterilized hollow steel tube, a 6 mm-diameter hole was created in the central region of each parietal bone. Subsequently, testing materials such as GGT and GGTELK scaffolds were placed on the area with the hole in direct contact, forming an organ culture unit. The organ culture unit was then transferred to a Fitton-Jackson modification of Bigger’s culture medium, supplemented with 10% fetal calf serum, 20 mL/mL 200 mM glutamine, 25 mL/mL HEPES 1 M solutions, 10 mL/mL b-glycerol-2-phosphate 1 M solution, and 50 mg/mL ascorbic acid.

For the antibacterial assessment, a concentration of 1 × 10^3^ colony-forming units per milliliter (CFU/mL) of *S. aureus* was introduced into the 6 mm hole. Following a 24 h incubation period, the cell densities of *S. aureus* were measured turbidimetrically using optical density at 600 nm and referenced against a standard curve. After a 2-day infection, assays for IL-6 were performed utilizing a commercially available enzyme-linked immunosorbent assay (ELISA) kit.

### 2.9. Statistical Analysis

The results are reported as the mean ± standard deviation. Statistical analysis was performed using Student’s t-test or one-way analysis of variance (ANOVA), followed by a post hoc Fisher’s least significant difference (LSD) test for multiple comparisons. The threshold for statistical significance was set at *p* < 0.05.

## 3. Results

### 3.1. The Antibacterial Effect of Lumbrokinase and Emodin Treatments

**Figure 1:**
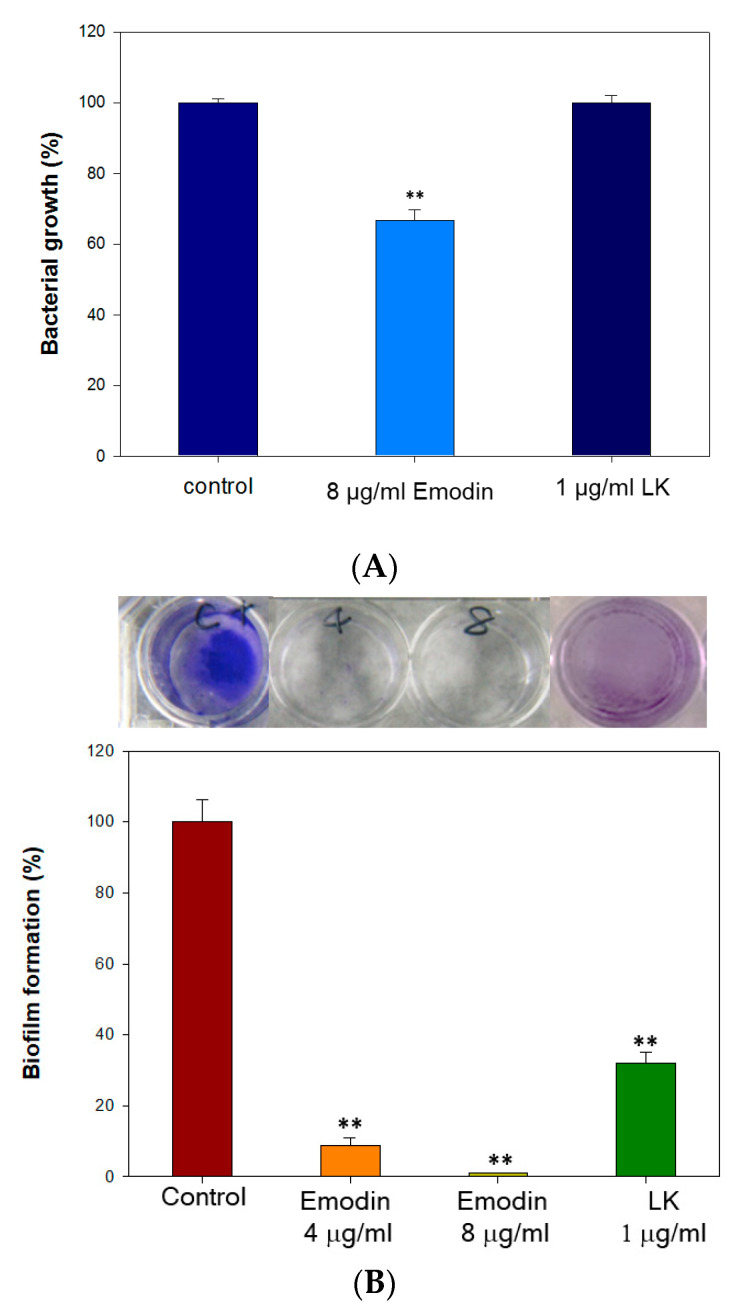
The antibacterial effect of lumbrokinase and emodin treatments. (**A**) The antibacterial growth rates of emodin and lumbrokinase against *S. aureus* (each well is 6 mm in diameter). (**B**) The inhibition rates of biofilm formation of emodin and lumbrokinase against *S. aureus.* (** indicates *p* < 0.01).

The results demonstrated that a concentration of 8 µg/mL of emodin effectively reduced bacterial growth by more than 2.8 log CFU/mL, which is considered sufficient to prevent bacterial proliferation (Table 1). In contrast, LK at a concentration of 1 µg/mL did not exhibit a significant impact on bacterial growth against *S. aureus* (Figure 1A). However, Emodin and LK demonstrated highly effective inhibition of biofilm formation (Figure 1B). At the concentration of 8 µg/mL and 1 µg/mL, emodin reduced biofilm formation by 100% and LK reduced it by 68%. The formation of biofilms is of utmost importance for bacterial survival and their ability to withstand challenging environments. Therefore, the inhibitory effects of emodin and LK on biofilm formation suggest their potential as suitable drugs against biofilm-associated infections.

Emodin exerts antibacterial activity by disrupting cell membrane integrity, increasing membrane permeability, and influencing membrane protein conformation [26]. Additionally, it disrupts the extracellular DNA release and suppresses the expression of genes linked to the formation of biofilms [31]. These findings support emodin as an effective drug for preventing *Staphylococcus aureus* biofilm-associated infections. LK also effectively inhibits biofilm formation through its fibrinolytic activity, which enables it to dissolve biofilms. The combined use of emodin and LK may exhibit the antibacterial effect through a different mechanism. The results of the antibacterial assay suggest that emodin, at a concentration of 8 µg/mL, has a significant bactericidal effect. In contrast, LK, even at a concentration of 1 µg/mL, does not demonstrate significant antibacterial activity against *S. aureus*. However, both emodin and LK exhibit strong inhibitory effects on biofilm formation. This highlights the potential of emodin and LK as effective antibacterial treatments for biofilm-related infections.

### 3.2. Effects of Emodin and Lubrokinase Treatment on Osteoblast Cells

#### 3.2.1. Lumbrokinase-Activated Osteoblast Differentiation

**Figure 2:**
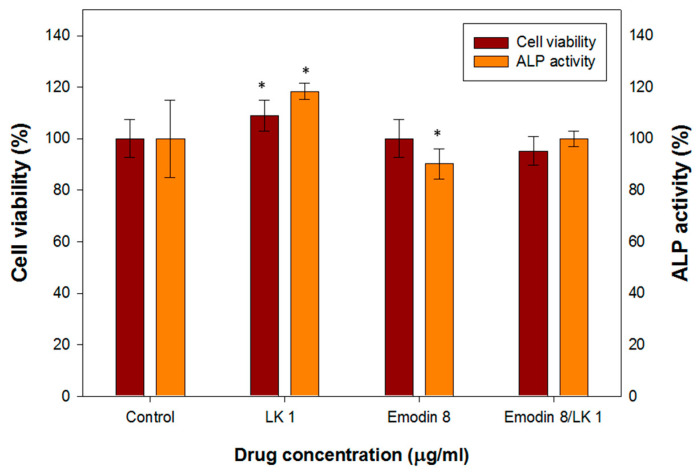
The cell viability and ALP activity assay of MC3T3-E1 cells treated with various concentrations of LK and emodin. (* indicates *p* < 0.05).

MC3T3-E1 cells were subjected to various concentrations of lumbrokinase (LK) and emodin, and their impact on cell viability was assessed using MTT assays. Additionally, the activity of ALP, an early marker of osteoblast differentiation, was examined. In Figure 2, the results demonstrate that a concentration of 1 µg/mL of LK led to an 8.9% increase in MC3T3-E1 cell viability and an 18.4% increase in ALP activity. These findings indicate that LK enhances the viability and differentiation of osteoblast cells. Similar effects have been observed in previous studies, where a concentration of 1 µg/mL of LK resulted in a 23% increase in MG-63 cell viability (a human osteoblast-like cell line) and a 7.4% increase in ALP activity, along with an upregulation of osteopontin expression [30]. These results suggest that LK may promote osteoblast viability and contribute to bone remodeling potential.

The determination of the optimal dosage of emodin is a critical aspect in the production of biomedical composites. This is particularly important due to the documented positive pharmacological effects of emodin on human health as well as its potential toxicity to various tissues or organs. Figure 2 illustrates the effects of different concentrations of emodin on osteoblast cell viability and ALP (alkaline phosphatase) activity. At a concentration of 8 µg/mL, emodin demonstrated minimal influence on the viability of osteoblast cells, indicating its relative safety within this range. However, it did cause a slight decrease in ALP activity. Previous studies have reported that emodin can stimulate the formation of osteoblasts in primary osteoblast cultures. However, the same effect was not observed in MC3T3-E1 cells, which are an osteoblast-like cell line.

Interestingly, when MC3T3-E1 cells were treated with a combination of 8 µg/mL of emodin and 1 µg/mL of LK (an unidentified compound), their cell viability and ALP activity remained comparable to those of the control group. This suggests that the addition of LK may compensate for the slight inhibition of ALP activity caused by emodin.

#### 3.2.2. Lumbrokinase-Increased Osteoblast Mobility

**Figure 3:**
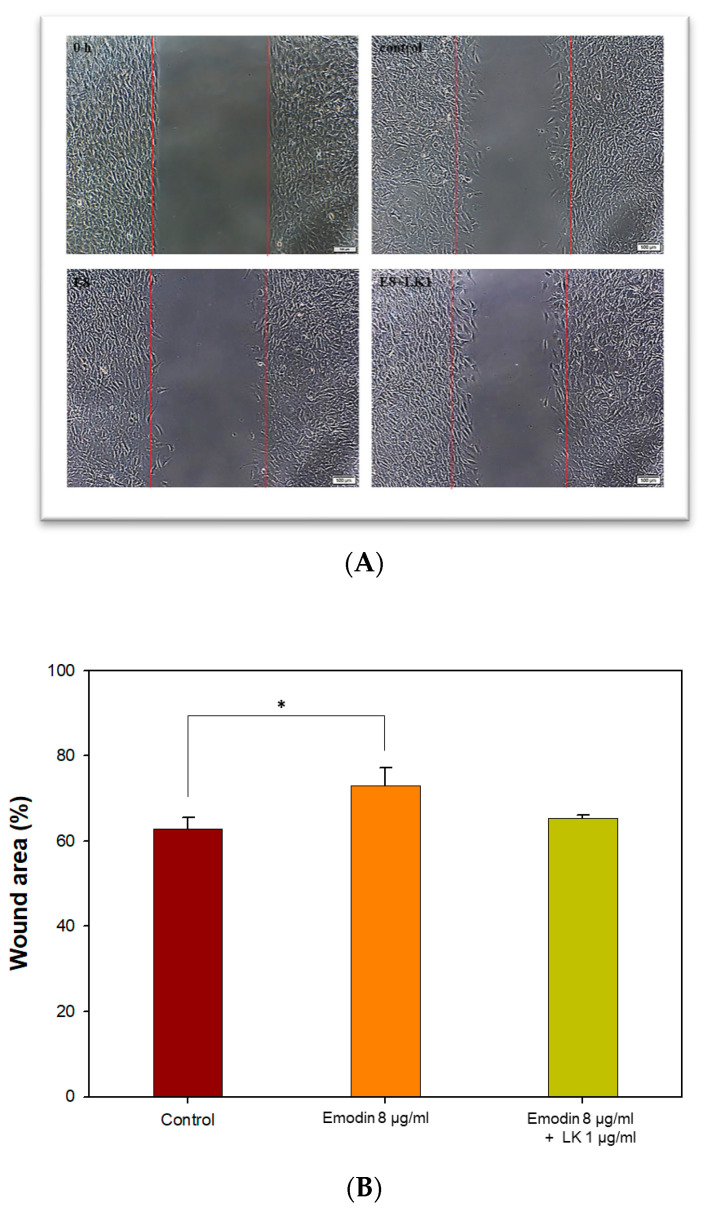
Effects of emodin/LK treatment on osteoblast migration. (**A**) Photomicrographs of cell migration were obtained two days after wounding. The white bars indicate 100 μm. (**B**) The relative proportion of wound area with different treatments. (* indicates *p* < 0.05).

The effect of emodin/lumbrokinase (LK) treatment on osteoblast migration was evaluated using an in vitro wound-healing assay. As mentioned, a concentration of 8 µg/mL of emodin demonstrated an excellent antibacterial effect, but it had minimal influence on MC3T3-E1 cell viability and slightly decreased alkaline phosphatase (ALP) activity. In the osteoblast migration assay, 8 µg/mL of emodin resulted in a 10% reduction in osteoblast mobility (Figure 3A,B). In contrast, previous research has shown that a concentration of 1 µg/mL of LK increased the migration of human osteoblast-like cells (MG-63) [23].

Therefore, in order to achieve an antibacterial and biocompatible GGT composite, the combination of emodin and LK proved to be the most favorable option. Moreover, the combination of 8 µg/mL of emodin and 1 µg/mL of LK appeared to be the most favorable option for promoting osteoblast migration. The addition of LK compensated for the decreased migration of MC3T3-E1 cells caused by the 8 µg/mL emodin treatment. This observation suggests a potential effect between emodin and LK in enhancing osteoblast migration, further emphasizing the importance of their combined application in biomedical composite development.

### 3.3. Effects of Emodin and Lubrokinase Treatment on Osteoclast Cells

#### 3.3.1. LK- and Emodin-Inhibited TRAP Activity Stimulated by RANKL in RAW264.7 Cells

**Figure 4:**
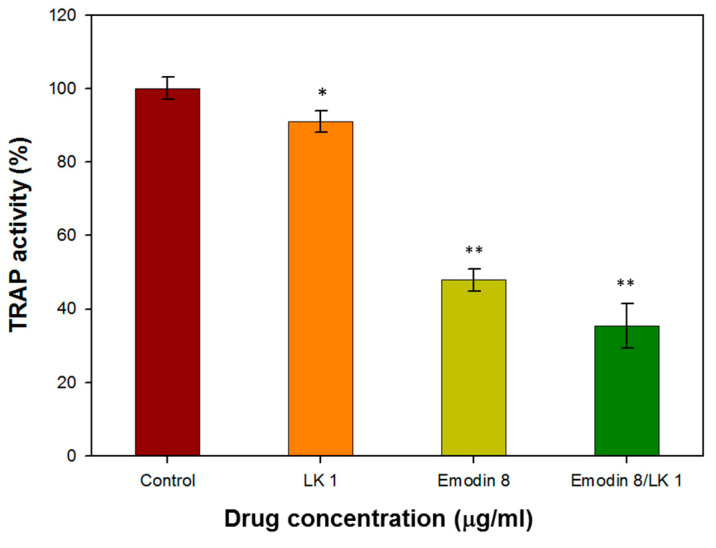
The TRAP activity of RAW264.7 cells treated with with 1 µg/mL LK and 8 µg/mL emodin. (* indicates *p* < 0.05; ** indicates *p* < 0.01).

Murine monocyte/macrophage RAM264.7 cells were employed to investigate the impact of lumbrokinase (LK) and emodin on osteoclasts. The impact on osteoclast differentiation was assessed through the utilization of the tartrate-resistant acid phosphatase (TRAP) activity assay, a crucial cytochemical marker that indicates osteoclast function and the extent of bone resorption. The TRAP activity of RAW264.7 cells was reduced by 9% and 52% following treatment with LK and emodin, respectively. Moreover, the presence of emodin and LK led to a significant inhibition (65% reduction) of RANKL-induced TRAP activity (Figure 4).

The inhibitory effect of emodin on osteoclast differentiation and bone resorption is consistent with previous studies that have reported its ability to suppress RANKL-induced NF-κB signaling and the expression of c-fos and NFATc1, which are key regulators of osteoclast differentiation [32]. These findings further support the notion that emodin can effectively interfere with the initial cellular events of osteoclast formation.

The combination of LK and emodin appears to offer a synergistic effect in inhibiting osteoclast function. While LK alone showed a modest reduction in TRAP activity, its combination with emodin resulted in a more pronounced inhibitory effect. This suggests that a combined therapy may have a stronger impact on osteoclasts than either compound alone.

#### 3.3.2. LK and Emodin-Decreased IL-6 Levels in RAW264.7 Cells

**Figure 5:**
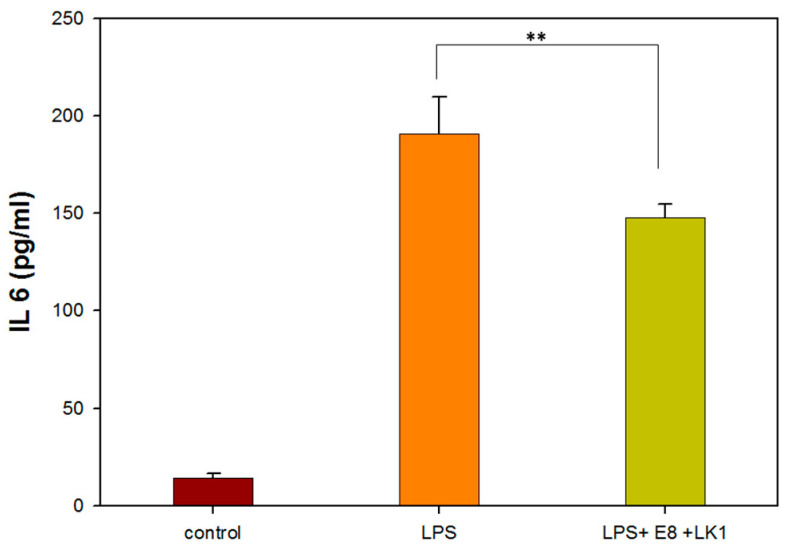
IL 6 assay of RAW264.7 cells treated with 1 µg/mL LK and 8 µg/mL emodin. (** indicates *p* < 0.01).

The levels of the pro-inflammatory cytokine IL-6 were assessed using a commercial ELISA kit after treating RAW264.7 cells with a concentration of 8 µg/mL emodin and 1 µg/mL lumbrokinase (E8/LK 1). In Figure 5, the IL-6 level was observed to decrease by 22.6% following treatment with 8 µg/mL emodin and 1 µg/mL lumbrokinase (E8/LK 1) in RAW264.7 cells. Moreover, the E8/LK 1 combination exhibited a strong inhibition of bacterial growth and biofilm formation while decreasing the levels of the pro-inflammatory cytokine IL-6. Importantly, the dual combination demonstrated good biocompatibility.

Interleukin-6 (IL-6) has been shown to have both pro-inflammatory and anti-inflammatory properties. Studies have demonstrated that an appropriate level of inflammation, characterized by a controlled release of pro-inflammatory cytokines such as IL-6, can facilitate bone tissue repair. Excessive production of IL-6 can lead to prolonged inflammation, which can impede the healing process and result in tissue damage. Therefore, controlling and modulating the levels of IL-6 can help ensure a balanced and controlled inflammatory response.

Previous pharmacological studies have demonstrated the anti-inflammatory effects of emodin, with a decrease in IL-6 levels. Furthermore, emodin treatment has been shown to inhibit TNF-α and IL-6 in the plasma of mice with collagen-induced arthritis [33]. Lumbrokinase treatment has also been reported to reduce oxidative damage, inflammation, and apoptosis induced by myocardial ischemia–reperfusion (I–R) through the activation of Sirt1 signaling [34]. Based on these findings, the E8/LK 1 combination was selected as the optimal choice for the manufacture of the antibacterial and anti-inflammatory GGTELK composite.

### 3.4. Characterization of GGT and GGTELK Scaffolds

#### 3.4.1. Cumulative Drug-Released Concentration and Degradation Rate of GGT

**Figure 6:**
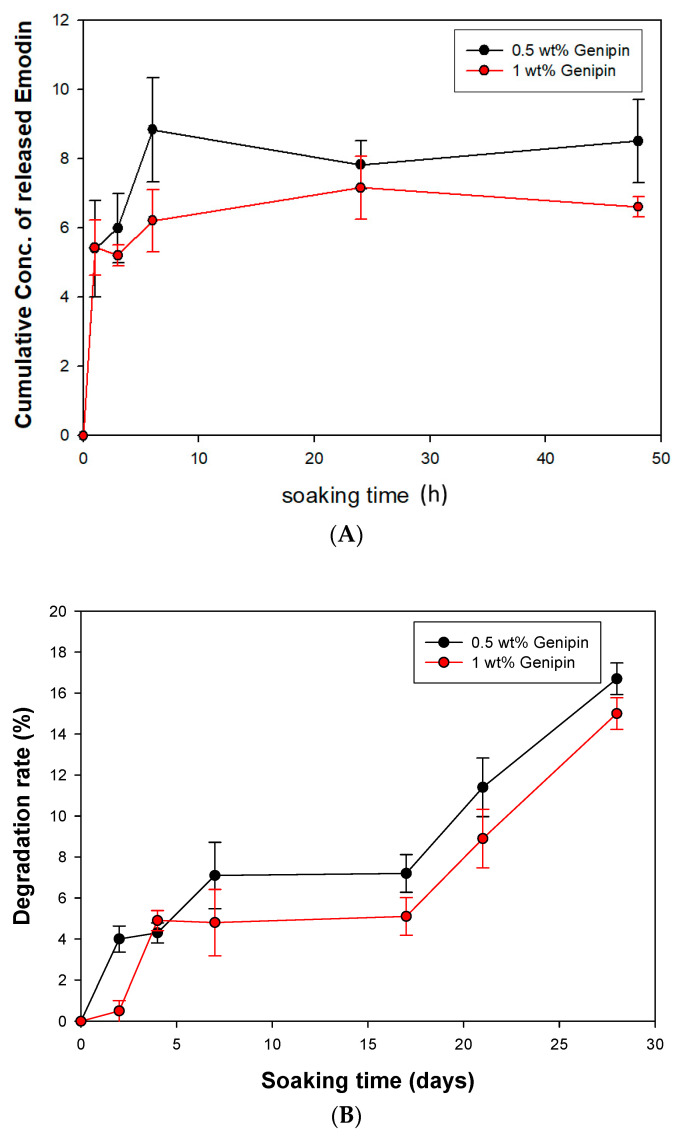
The cumulative drug-released assay and degradation rate of GGT (**A**) Cumulative drug-released concentration of GGTE scaffolds. (**B**) Degradation rates of GGT composites.

The controlled release of bioactive compounds is crucial for promoting bone regeneration while maintaining structural stability. The gelatin complex mixed with tricalcium phosphate ceramic particles (GGT) was crosslinked with genipin and loaded with emodin to create a functional GGTE composite. Various concentrations (0.5 wt% and 1 wt%) of genipin were examined to ensure the production of a biomechanically stable composite. In Figure 6A it was observed that for GGTE crosslinked with 0.5 wt% genipin after soaking for 6 h, the cumulative concentration of emodin reached 8.8 µg/mL, effectively inhibiting the growth of microorganisms. The GGT crosslinked with 1 wt% genipin required 96 h to release an equivalent amount of emodin. Since the drug release assay of GGTLK has already been reported in our previous results [23], we did not replicate this assay for LK.

While GGT possesses interconnected porous structures that are beneficial for fibrovascular invasion and bony replacement, it may also compromise the mechanical properties [16]. To assess the degradation rates and evaluate the biochemical stability, the GGT composites were soaked for 28 days. As shown in Figure 6B, the 28-day degradation rates of GGT crosslinked with 0.5 wt% and 1 wt% genipin were found to be 16.72% and 14.95%, respectively. These results indicate that the composite can provide both stability and an adequate timeframe for bone recovery.

The results demonstrate the successful development of a GGTE composite with controlled release of emodin. The selection of genipin concentration for crosslinking influenced the release rate of emodin, with 0.5 wt% genipin achieving a quicker release within 6 h. The degradation rates of both composites were within an acceptable range, ensuring an adequate timeframe for bone recovery.

#### 3.4.2. Biocompatibility of GGTELK

**Figure 7:**
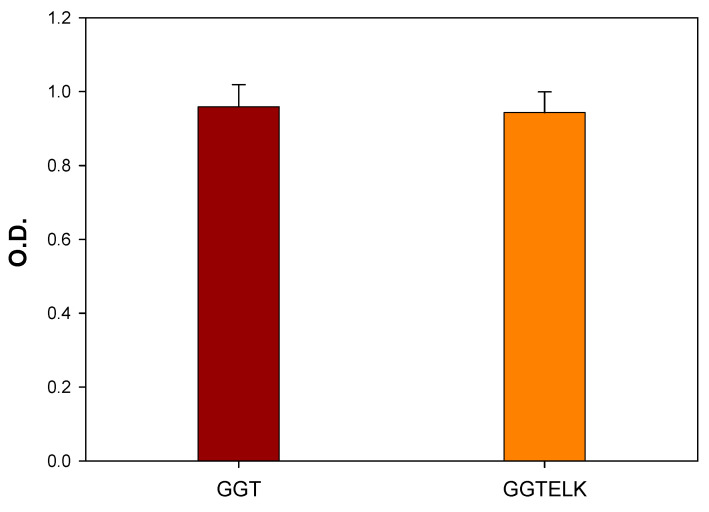
Cell viability assay of MC3T3-E1 cells co-cultured with GGT and GGTELK.

MC3T3-E1 cells were co-cultured with the immersion solutions of GGT and GGTELK to assess cell viability. Following the two-day incubation, the MTT assay was conducted to evaluate any potential cytotoxic effects resulting from the addition of emodin and LK into GGT. As shown in Figure 7, the results indicated that the inclusion of emodin and LK in GGT did not exhibit any cytotoxicity towards MC3T3-E1 cells. These findings are promising as they indicate that the incorporation of emodin and LK in GGT does not compromise the overall viability of the MC3T3-E1 cells. This supports the potential use of the composite materials in tissue engineering applications as they can interact with cells without causing any significant cytotoxic effects.

#### 3.4.3. Antibacterial Effect of GGTELK

**Figure 8:**
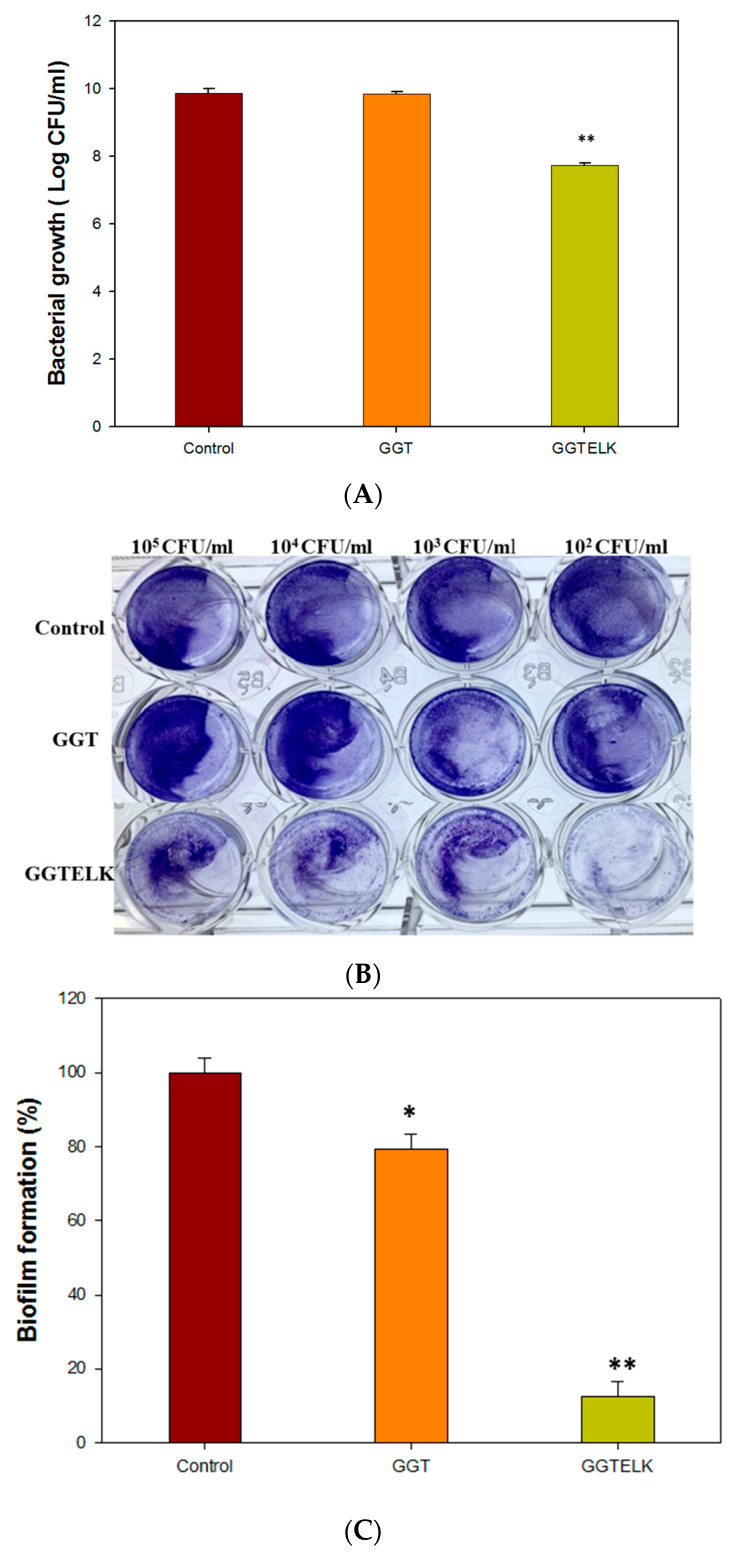
Antibacterial effect of GGTELK scaffolds. (**A**) The growth of *S. aureus* after 24 h incubation with GGT or GGTELK scaffolds, respectively. (**B**) The biofilm formation of *S. aureus* cultured with GGT and the GGTELK scaffolds at various cell densities (each well is 6 mm in diameter). (**C**) Effect of GGTELK on biofilm formation of *S. aureus* with GGT and the GGTELK scaffolds at 1 × 10^5^ CFU/mL (* indicates *p* < 0.05; ** indicates *p* < 0.01).

The evaluation of the antibacterial and anti-biofilm properties of biomaterials is crucial for their application in combating bacterial infections. To evaluate the antibacterial properties of GGTELK, GGT and GGTELK scaffolds were co-cultured with *S. aureus* at a cell density of 1 × 10^5^ CFU/mL. After a 24 h incubation period, the results demonstrated that the GGTELK scaffold effectively reduced the growth of *S. aureus*, as shown in Figure 8A. The reduction in bacterial growth exceeded 2.1 log CFU/mL, indicating a significant antibacterial effect. Notably, the antibacterial efficacy of GGTELK was comparable to that of 8 µg/mL of emodin, highlighting the potent antibacterial activity of the GGTELK composite.

The inhibitory effects of emodin and lumbrokinase (LK) on *S. aureus* biofilm formation were evident. Moreover, the GGTELK scaffolds demonstrated remarkable anti-biofilm properties when co-cultured with *S. aureus* at various cell densities, as shown in Figure 8B. Notably, at a low cell density of 1 × 10^2^ CFU/mL, the GGTELK scaffolds exhibited significant inhibition of biofilm formation. Additionally, in Figure 8C, the GGTELK scaffolds cultured with *S. aureus* at a cell density of 1 × 10^5^ CFU/mL achieved an impressive 88% reduction in biofilm formation.

The evaluation of GGTELK demonstrated its significant antibacterial activity against *S. aureus* and its effective inhibition of biofilm formation. The potent antibacterial and anti-biofilm properties of GGTELK, attributed to the inclusion of emodin and LK, suggest its potential as a promising biomaterial for combating biofilm-related infections.

#### 3.4.4. Antibacterial and Anti-Inflammatory Effects of GGTELK Assessed in an Ex Vivo Bone Defect Model

**Figure 9:**
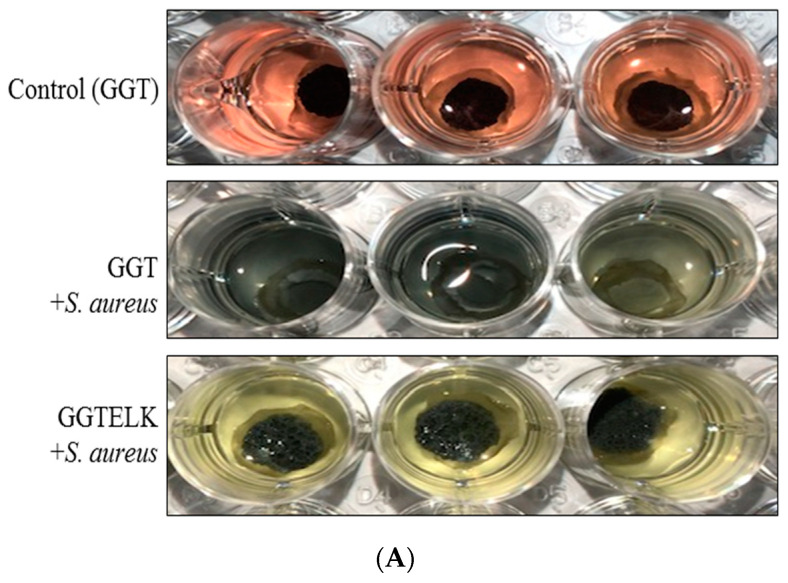

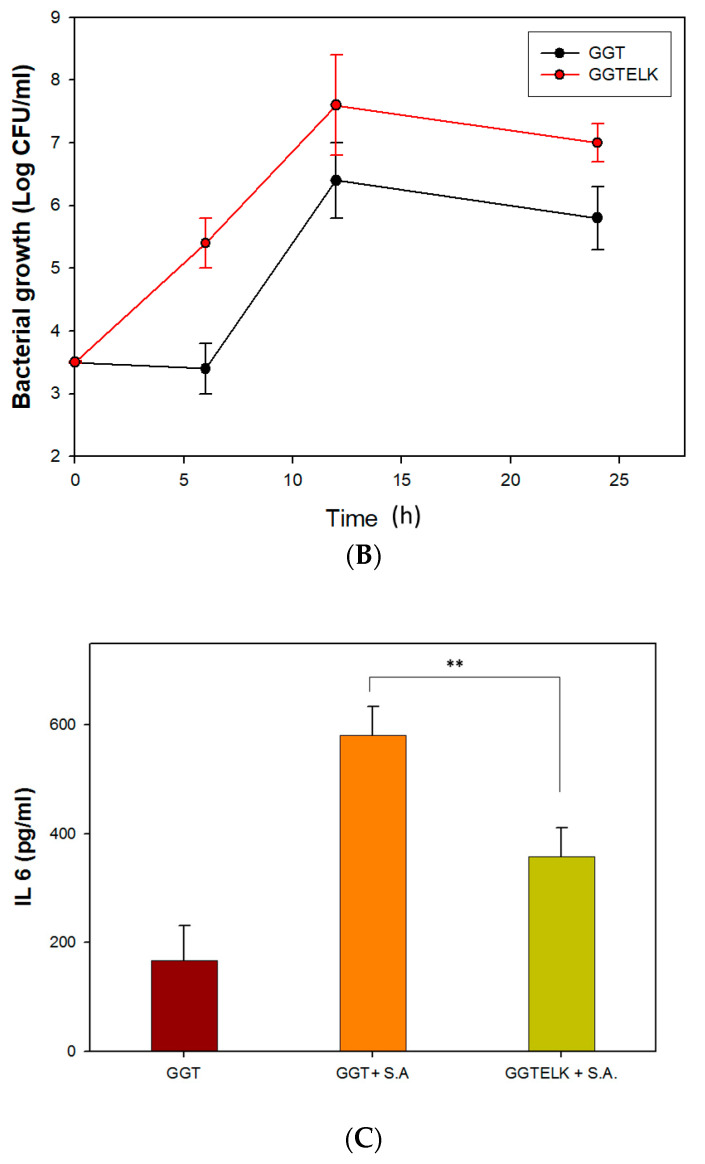
The antibacterial and anti-inflammatory effects of GGTELK in an ex vivo bone defect model. (**A**) Photographs of antibacterial assays of GGT and GGTELK scaffolds in ex vivo models (each well is 16 mm in diameter). (**B**) Bacterial growth of *S. aureus* in ex vivo models co-cultured with GGT and GGTELK scaffolds. (**C**) The IL-6 secretion induced by *S. aureus* infection in ex vivo models co-cultured with GGT and GGTELK scaffolds. (** indicates *p* < 0.01).

To assess the antibacterial and anti-inflammatory effects of GGTELK, an ex vivo model was employed as an alternative to in vivo animal models to minimize the influence of environmental and physiological factors. In this model, GGT and GGTELK scaffolds were placed within a 6 mm defect area in calvarial bone. Subsequently, *S. aureus* at a concentration of 1 × 10^3^ CFU/mL was introduced, and the cultures were maintained for 24 h. The bacterial growth was quantified using a turbidometric assay at 600 nm. The OD600 nm readings were recorded as 7.0 and 5.8 for the GGT and GGTELK scaffold groups, respectively (Figure 9A,B). Notably, the drug release assay demonstrated that the cumulative concentration of emodin released by the GGTELK scaffolds reached 8.8 µg/mL, effectively inhibiting bacterial growth. Furthermore, in Figure 9B, the growth rate of *S. aureus* was completely suppressed during the initial 6 h period in the GGTELK scaffold groups. Moreover, following *S. aureus* infection, the secretion of IL-6 from the calvaria was reduced by 38.4% compared to the GGT group (Figure 9C).

The utilization of the ex vivo model demonstrated that GGTELK scaffolds effectively inhibited bacterial growth and exhibited anti-inflammatory properties. The release of emodin and LK from the scaffolds contributed to their antibacterial effect, as indicated by the suppression of *S. aureus* growth. Moreover, the reduction in IL-6 secretion reflected the anti-inflammatory properties of the GGTELK scaffolds. These results indicate that the GGTELK scaffolds released sufficient antibacterial and anti-inflammatory components, effectively inhibiting bacterial growth and demonstrating their antibacterial effect in the ex vivo model.

## 4. Conclusions

The GGT scaffolds augmented with emodin and lumbrokinase exhibited notable antibacterial and anti-inflammatory effects in both in vitro assays and ex vivo models. Emodin demonstrated potent antibacterial and anti-inflammatory properties, while lumbrokinase effectively inhibited biofilm formation and positively influenced bone cell culture. As a result, the GGTELK composite, characterized by stability, efficient degradation, biocompatibility, and anti-inflammatory functionality, shows promise as an ideal bone substitute. The combination of these bioactive components enhances the therapeutic potential of the composite, making it a promising candidate for bone regeneration applications. Moreover, based on the excellent antibacterial characteristics of emodin, its application in biomedical material shows promising potential for future studies.

## Figures and Tables

**Table 1:** The antibacterial growth of emodin and lumbrokinase against *S. aureus*.

Log (CFU/mL)	0 h	24 h
Control	5.6 ± 0.2	9.6 ± 0.1
8 µg/mL Emodin	5.6 ± 0.2	6.4 ± 0.3
1 µg/mL Lumbrokinase	5.6 ± 0.2	9.5 ± 0.2

## Data Availability

Not applicable.
